# Training nurse simulation educators at scale to improve maternal and newborn health: a case study from Bihar, India

**DOI:** 10.1186/s12909-022-03911-9

**Published:** 2022-12-15

**Authors:** Solange Madriz, Patience Afulani, Hilary Spindler, Rakesh Ghosh, Nidhi Subramaniam, Tanmay Mahapatra, Aritra Das, Sunil Sonthalia, Aboli Gore, Susanna R. Cohen, Seema Handu, Dilys Walker

**Affiliations:** 1 Institute for Global Health Sciences, University of California, San Francisco, San Francisco, California USA; 2 Department of Epidemiology and Biostatistics, University of California, San Francisco, San Francisco, California USA; 3 PRONTO International, Patna, Bihar India; 4 CARE India, Patna, Bihar India; 5 Department of Obstetrics and Gynecology University of Utah, Salt Lake City, UT USA

**Keywords:** Simulation, Health Education, Global Health, Simulation Educator, Maternal and Newborn Health, Low-resource setting

## Abstract

**Background:**

Simulation has been shown to improve clinical and behavioral skills of birth attendants in low-resource settings at a low scale. Populous, low-resource settings such as Bihar, India, require large cadres of simulation educators to improve maternal and newborn health. It’s unknown if simulation facilitation skills can be adopted through a train of trainers’ cascade. To fill this gap, we designed a study to evaluate the simulation and debrief knowledge, attitudes and skills of a third generation of 701 simulation educators in Bihar, India. In addition, we assessed the physical infrastructure where simulation takes place in 40 primary healthcare facilities in Bihar, India.

**Methods:**

We performed a 1 year before–after intervention study to assess the simulation facilitation strengths and weaknesses of a cadre of 701 nurses in Bihar, India. The data included 701 pre-post knowledge and attitudes self-assessments; videos of simulations and associated debriefs conducted by 701 providers at 40 primary healthcare centers.

**Results:**

We observed a statistically significant difference in knowledge and attitude scores before and after the 4-day PRONTO simulation educator training. The average number of participants in a simulation video was 5 participants (range 3-8). The average length of simulation videos was 10:21 minutes. The simulation educators under study, covered behavioral in 90% of debriefs and cognitive objectives were discussed in all debriefs.

**Conclusion:**

This is the first study assessing the simulation and debrief facilitation knowledge and skills of a cadre of 701 nurses in a low-resource setting. Simulation was implemented by local nurses at 353 primary healthcare centers in Bihar, India. Primary healthcare centers have the physical infrastructure to conduct simulation training. Some simulation skills such as communication via whiteboard were widely adopted. Advanced skills such as eliciting constructive feedback without judgment require practice.

## Background

Simulation training is the recreation of a real-life scenario for the purpose of practicing and learning from the behaviors realized in response to the reenactment event. In high and middle-income countries, simulation training is widely accepted as an important educational methodology to develop, sustain, and improve clinical and interprofessional skills of pre- and in-service professionals [1]. Given the experiential nature of this learning methodology, simulation is thought to lead to longer sustained improvements in knowledge and skills compared to traditional methods [2]. Simulation has been particularly useful for practicing appropriate management of clinical emergencies, as simulated scenarios recreate the stress of a real-life event without the consequences of improper management during an actual case [3]. The successes observed in middle and high-income countries have encouraged efforts to adapt simulation trainings to lower resource settings using lower-technology tools to decrease the costs of implementation [4, 5]. Some simulation-based training programs have been implemented at small to medium size scale to build capacity and improve quality of maternal and newborn health in low- resource settings [6]. However, if this approach is to contribute to ongoing improvement in maternal and newborn indicators, we need to ensure a sustainable and well-trained cadre of simulation educators in low-resource settings to implement this important strategy.

**Fig. 1:**
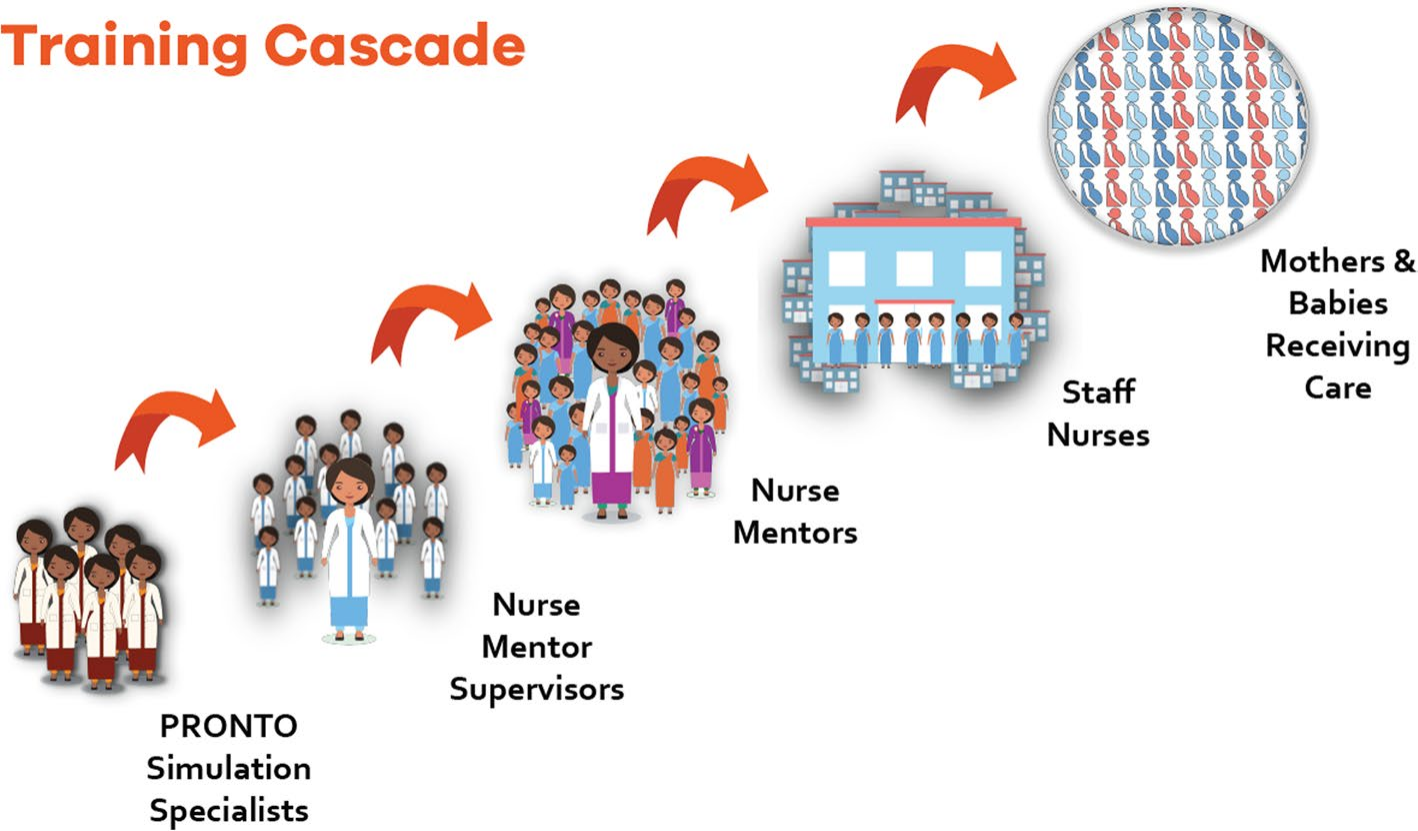
Cascade of simulation educators during AMANAT-Jyoti program in Bihar, 2018 onwards

With a population of about 120 million, a maternal mortality rate of 149 per 100,000 live births and neonatal mortality rate of 25 per 1000 live births, the state of Bihar has maternal and neonatal mortality rates higher than the national average [7, 8]. Bihar is India’s third most populated state and one of the poorest regions in India and South Asia [9]. Increasing births in healthcare facilities has not improved the outcomes, due in large part to a shortage of skilled birth attendants in public healthcare facilities [10]. With a rising number of institutional births in this densely populated state, the demand for skilled birth attendants to provide high quality care has accelerated. To address this quality of care gap, the government of Bihar and their main collaborators, CARE India, and the Bill and Melinda Gates Foundation, launched a pilot program called the Mobile Nurse Mentoring Team (MNMT) in 8 districts of Bihar. In 2015, the MNMT was scaled up into the state-wide AMANAT program. AMANAT which stands for “*Aapatkalin Matritva Avam Navjaat Shishu Tatparta*” in Hindi, Emergency Preparedness for Maternal, Newborn and Baby was a nurse mentoring program for facility and auxiliary nurse midwives (ANM) to improve management of obstetric and newborn complications. As part of the MNMT (2012 – 2015) and the AMANAT (2015 – 2017) programs, formally trained (bachelor of science or master of science in nursing) nurse mentors (NM) provided onsite mentoring to the nurses and the ANM posted in the labor rooms and maternity wards at the Basic Emergency Obstetric and Newborn Care (BEmONC) and Comprehensive Emergency Care Obstetric and Newborn (CEmONC) public healthcare facilities excluding the medical colleges, across the state of Bihar. The NM incorporated diverse teaching strategies including on-the-job demonstrations of evidence-based practices on actual patients (antenatal and postnatal women and the newborns), bedside mentoring, didactics, and high fidelity, low-cost simulation and team-training program called PRONTO™. Using simulation, debriefs and team training activities, PRONTO’s program is focused on improving the management of the most common maternal and newborn emergencies. The AMANAT program was effective in improving evidence-based practices in normal as well as complicated births [11] however, given that the nurse mentors were ‘external’ to the system (i.e., they were employed by Bihar Technical Support Program but were not part of the state’s public health system), the program was neither financially nor realistically sustainable. Thus, to bolster the health gains obtained with the AMANAT program and to make the program more affordable and sustainable, the government of Bihar and their collaborators launched the AMANAT-Jyoti program in 2018. The AMANAT-Jyoti Nurse Mentoring Program, a capacity strengthening program led by CARE India deployed in 353 facilities, comprised of 331 BEmONC and 22 CEmONC level public health facilities across the state of Bihar, India focused on improving the quality of obstetric and newborn care through onsite mentoring of, and by, facility-based nurses and ANMs. During this iteration of the program, over 700 AMANAT-trained government nurses and ANMs working at the public facilities were selected to become ‘nurse mentors’ in their own facilities. These nurse mentors were responsible for training the facility nurses/ANMs on all training modules including PRONTO simulation and team training. During this phase, a third cadre of off-site ‘nurse mentor supervisors’ (NMS) were added to provide support to the facility-based nurse mentors. This study assessed the simulation and debriefing knowledge, attitudes self-assessment, and skills of the third generation of simulation educators (the AMANAT Jyoti NMS) and described the characteristics and physical conditions in which simulation and debriefs took place in 40 public healthcare facilities in 2018. To our knowledge, this is the first study documenting the simulation and debriefing skills of a cadre of nurse mentors trained in simulation facilitation in such a training of trainer cascade (Fig. 1).

## Methods

### Intervention

The AMANAT-Jyoti Nurse Mentoring Program curriculum included multiple modules on evidence-based ante-, intra- and post-partum care and neonatal-care practices and a locally tailored library of simulation scenarios on the most common obstetric and neonatal emergencies such as post-partum hemorrhage, eclampsia, and neonatal resuscitation as well as teamwork and communication activities to strengthen effective communication, leadership and teamwork and kind and respectful care. PRONTO International, the University of California San Francisco and the University of Utah were partner collaborators for the simulation and team-training component of the AMANAT-Jyoti program.

To facilitate scale-up and sustainability, the AMANAT-Jyoti program featured three generations of simulation educators (Fig. 1). The first were the Nurse Mentor Supervisors (NMS) who have a bachelor’s degree in nursing and a majority of them were residents of states other than Bihar. Sixty NMS participated in a six-day Simulation Educator Training (SET) conducted in January 2018 by PRONTO International. The SET was held in Patna, the state capital of Bihar, and was led by six PRONTO master trainers in conjunction with local staff members. The master trainers were five local nurses with advanced degrees in nursing and one foreign midwife educator from the United States. The NMS were trained in how to teach others to facilitate 7 different simulation scenarios and 7 teamwork and communication activities based on the AMANAT-Jyoti learning objectives. The third generation of educators were the AMANAT-Jyoti Nurse Mentors (NM), who have an equivalent of 1-2 years of nursing training providing care in public health facilities with 24-hour obstetric care. AMANAT-Jyoti NM were participant-trainees of the previous iteration of the AMANAT mentoring program and were selected using standardized test of clinical knowledge and Objective Structured Clinical Examinations. The selected 701 AMANAT-Jyoti NM attended a four-day SET from March – April 2018. A total of sixty SETs were conducted across the state of Bihar for AMANAT-Jyoti NM. The SETs were led by the 60 NMS who were previously trained by PRONTO International’s master trainers. The curriculum of the SET included components of a pre-brief, how to set up, facilitate and debrief seven obstetric and neonatal emergency simulation scenarios and seven teamwork and communication activities. The simulation setup curriculum included how to make simulated blood, dress, and prepare the simulated patient for a realistic scenario progression as well as on how to use the PartoPants™, the birth simulator worn by the simulated patient. The facilitation curriculum described the use of hand signals to communicate with the simulated patient during the scenario and the use of a whiteboard to indicate vital signs. The debrief model taught was the “Diamond Structure” which follows description, analysis, and application phases [12]. The debriefs of the intervention were done in a separate space as much as logistically possible. When it was not logistically possile, participants moved away from simulation space and sat in a semi-circle along with the debriefer, such that they were not facing the simulation area.

In addition, AMANAT-Jyoti NM learned how to debrief technical, cognitive, and behavioral elements during the analysis phase of the debrief. After the SET, AMANAT-Jyoti NM were assigned to work in pairs, supported by a NMS, to conduct simulation and teamwork and communication activities in their facilities.

### Study design

The data for this analysis came from knowledge and attitudes self-assessment questionnaires administered to all 701 AMANAT-Jyoti NM before and after the simulation educator training, as well as videos of simulations and associated debriefs conducted by the AMANAT-Jyoti NM at a subset of 40 public healthcare facilities. We had previously randomly selected a subset of 105 BEmONC and CEmONC level facilities for a different evaluation, and therefore selected 40 facilities from that subset for this study. The facilities whose videos were included in the analysis belonged to 16 districts out of a total of 38 districts (42%) in the state of Bihar. Video recordings of one simulation and its corresponding debrief were coded from each of the forty facilities. The videos were all taken from the first module of the AMANAT-Jyoti program which focused on the management of the most common obstetric and neonatal emergencies, post-partum hemorrhage, eclampsia and neonatal resuscitation, as well as respectful maternity care (RMC).

### Measurement tools

Training participants completed a knowledge test and attitudes self-assessment questionnaires with the same questions before and after the training using paper-based forms, in Hindi. The objective of these tools was to measure changes in simulation and debrief facilitation knowledge and attitudes as a result of attending the simulation educator training. The knowledge test included seventeen questions on simulation facilitation and teamwork and communication concepts. The attitudes self-assessment questionnaire was based on Bandura’s self-efficacy theory [13] and contained 10 statements on the respondent’s belief in their capacity to conduct a simulation. The attitudes self-assesment questionnaire used a four-point Likert scale (‘strongly disagree’, ‘disagree’, ‘agree’, and ‘strongly agree’).

**Fig. 2:**
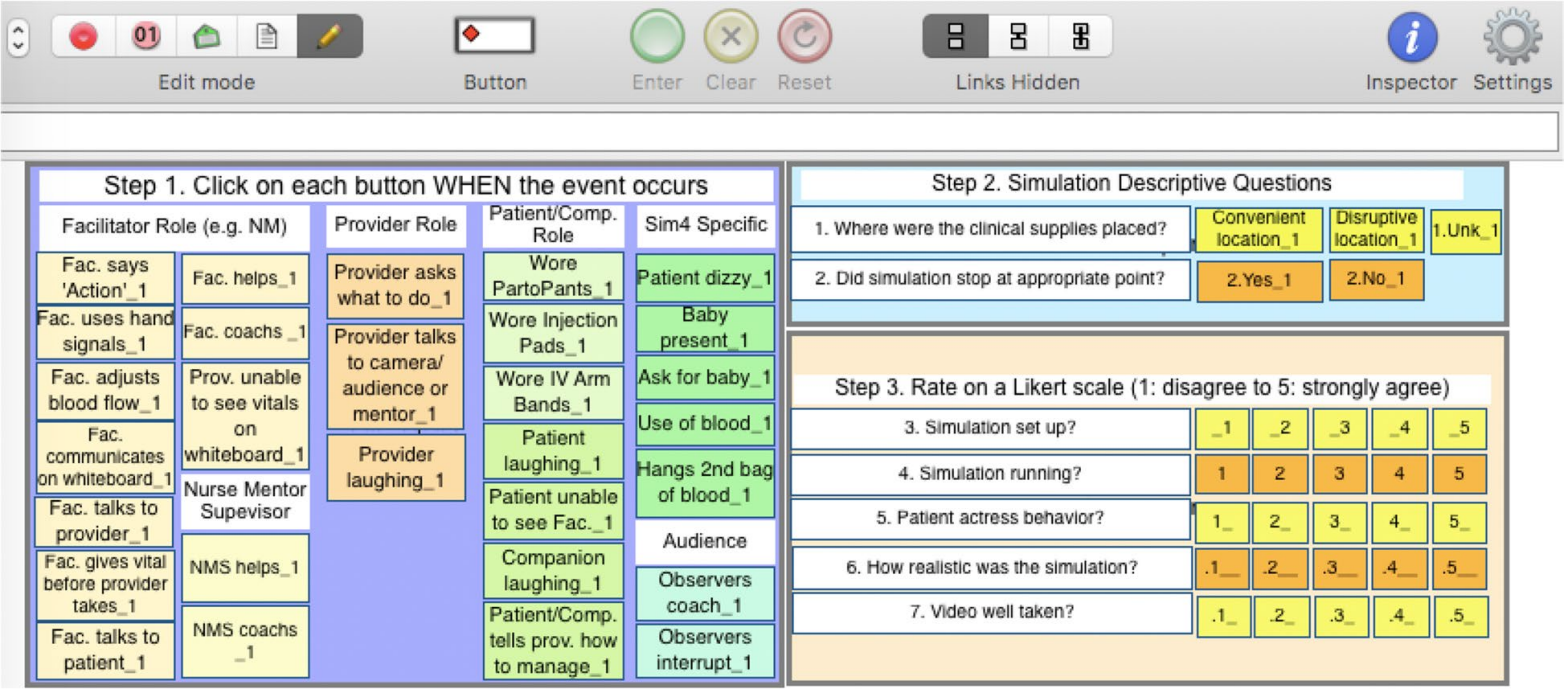
Code window for simulation video analysis

**Fig. 3:**
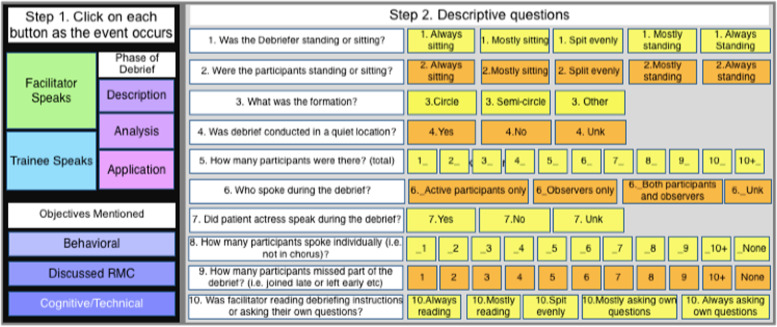
Code window for debrief video analysis

In addition to the paper-based assessments, we included simulation and debrief videos to assess AMANAT-Jyoti facilitation skills and abilities. The simulation scenario chosen for analysis was a case of postpartum hemorrhage due to uterine atony. Simulation and debrief videos were obtained from simulations conducted in each clinic over a three-month period from May to July 2018. Using Studiocode™, a team of simulation experts from UCSF, PRONTO International and University of Utah developed two code windows; one for the simulation and one for the corresponding debrief. The simulation code window included 35 indicators of best and negative practices to measure scenario setup, presence and use of simulation materials, instructions delivery, communication, progression of scenario, behavior of educator, participants, simulated patient and audience and overall scenario rating (Fig. 2). Educator, companion, and patient actress laughing were added as behavioral indicators in the simulation code window because it can signal either intense discomfort or inability to buy into the reality contract that is believing the simulation is real. Therefore, when doing data analysis laughing was included as a negative indicator. Observers’ behavior was also included in the simulation code window as PRONTO training emphasizes the role of the observers. Educators are taught to tell observers they cannot offer any help to the simulation participants. The debrief code window included eighteen indicators (Fig. 3) measuring time of educator speaking versus participants, number of participants that spoke, order of the debrief, type of objectives covered and sitting formation of participants. Both simulation and debrief code windows reflected the objectives of the scenario as well as simulation educator standards of best practices adapted to novice simulation educators [14, 15].

### Data analysis

The unit of analysis was a AMANAT-Jyoti nurse mentor. For the knowledge and attitudes self-assessment data, after the trainings were completed, the paper-based forms were entered into an online portal using Qualtrics (www.qualtrics.com) by members of the study team based in Patna. There were 684 (97.7%) participants that completed both forms at baseline while 693 (99%) participants completed both forms at endline. We conducted a bivariate analysis on the data using STATA 16 comparing the data from pre- to post-training. We estimated mean knowledge scores of individual questions pre- and post-training. A paired analysis was not feasible given the anonymous nature of the data collection. Thirty-nine simulation videos and 40 debrief videos were coded. The discrepancy was due to one corrupted simulation video which could not be recovered. The pre-post knowledge was unidentified and therefore we were not able to link it to the simulation and debrief video data. A team of two Hindi-speaking experienced video analysts based in the Patna office coded the videos from September to October 2018. Two simulation educators, trained as video analysts conducted the video review. One video analyst coded 34 pairs videos and the second video analyst coded six pairs videos. The videos were not double coded. A video database manager then exported the data to Microsoft Excel for analysis.

## Results

All the 60 NMS trained were female with an average age of 25 years (SD = 2, range: 21 - 34 years). Ninety-two percent (*n* = 55) had a Bachelors degree in nursing and 7% had a Masters degree in nursing. However, 60% (*n* = 55) had no prior experience working in a maternity ward. Also 42% (*n* = 25) reported they have had no prior training on patient-provider interaction and 76% (*n* = 47) had no prior training in simulation. Most (92%, *n* = 55) said they were either satisfied or very satisfied with their job. All the 693 AMANAT- Jyoti NM trained were female with an average age of 42 years (*N* = 631, SD = 9, range: 23 - 68 years).

The mean pre and post training knowledge was calculated from knowledge results for AMANAT-Jyoti NM presented in Table 1, showing there was a significant increase in mean knowledge between pre- and post- 4-day simulation facilitation training. The average knowledge score increase was from 49% at baseline to 65% at endline—a 16%-point difference (*p* < .001). Knowledge scores increased on all questions related to simulation facilitation and teamwork and communication except for one question where there was a slight decrease on the question related to the ‘role of educator as someone who guides participants to learn’.

**Table 1:** Simulation and debrief facilitation knowledge scores pre- and post- training for AMANAT-Jyoti nurse mentors in Bihar, India in March and April 2018, (*N* = 693)

Question Content	Pre (*n* = 684)	Post (*n* = 693)	Difference
1. Characteristic of a simulation educator	67%	62%	−5%
2. Information provided to participants during the pre-brief	69%	82%	13%
3. Participants who should hear the information about the patient scenario	52%	57%	5%
4. Use of whiteboard during simulation	62%	93%	31%
5. Use of baby cry during a neonatal resuscitation scenario	45%	49%	4%
6. Arrangement of participants during a debrief	46%	68%	22%
7. First question to be asked during a simulation debrief	48%	75%	27%
8. Example of an open-ended question used in a debrief	26%	34%	8%
9. Benefits of using open-ended questions	55%	58%	3%
10. Who should talk more during a debrief	51%	60%	9%
11. Benefits of debriefing a simulation scenario	51%	59%	8%
12. Thinking out loud concept	41%	74%	33%
13. Check-back concept	48%	81%	33%
14. Two challenge rule concept	57%	83%	26%
15. Appropriate use of SBAR	27%	46%	19%
16. Elements of good leadership	54%	60%	6%
17. First step before running a teamwork and communication activity	26%	55%	29%
Average:	49%	64%	15%

Attitudes also increased after the simulation educator trainings (Table 2). The average attitudes self-assessment score for overall simulation facilitation increased from 2.83 before the training to 3.43 after the training (*p* < .001). Results were measured on a four-point scale. On average, attitudes related to statements about mentoring increased from 3.03 before the training to 3.30 after the training (*p* < .001).

**Table 2:** Simulation facilitation attitudes self-assessment 4-point Likert scale scores pre- and post- training for AMANAT-Jyoti nurse mentors in Bihar, India in March and April 2018, (*N* = 701)

		Pre (*n* = 684)	Post (*n* = 693)	Difference
Category	Indicator	Mean (SD)		
Simulation Facilitation	I am able to setup a simulation.	2.8 (0.8)	3.5 (0.5)	0.6
	I am able to facilitate simulation.	2.7 (0.8)	3.4 (0.5)	0.7
	I am able to debrief a simulation.	2.6 (0.8)	3.4 (0.6)	0.8
	I am able to run a teamwork activity.	2.7 (0.8)	3.3(0.6)	0.6
	I am able to teach the communication techniques.	2.6 (0.9)	3.2 (0.6)	0.6
	Simulation training is important for the nurses and doctors in my clinic.	3.3 (0.8)	3.5 (0.5)	0.1
Mentoring	I am confident that I am able to teach emergency management using simulation.	3.19 (0.7)	3.53 (0.5)	0.3
	A mentor cannot change the way a nurse provides care.	2.52 (0.9)	2.60 (1.0)	−0.08
	If I try really hard I can give information to other nurses that will make their practice better.	3.4 (0.7)	3.61 (0.5)	0.2
	I know how to provide support and encouragement to students when I am leading a simulation.	3.0 (0.8)	3.46 (0.6)	0.4

Notes: Attitudes self-assessment scale ranged from strongly disagree to strongly agree

### Video analysis

The average length of the 39 simulation videos was 10.2 minutes (range: 2.3 – 23.4 minutes). The average number of participants was 5 (range: 3 – 9 participants) per simulation. The nurses participated in the scenario by playing different roles such as the nurse, patient and family members. The average length of the forty debrief videos was 12.5 minutes (range 5.3 – 28 minutes), with an average of 8 nurse participants (range: 3 – 13) per debrief.

### Environment

Most of the simulations were conducted in classrooms (*n* = 26, 65%), some were conducted in ‘other’ locations (*n* = 8, 20%), a few were conducted in the operation theater (*n* = 5, 13%) and one was conducted in the labour room. Most of the simulations were done in a quiet location (*n* = 28, 70%), some were done where there was some noise (*n* = 7, 18%) and a few were done in noisy locations (*n* = 5, 13%). The majority of simulations were conducted in locations with good light (*n* = 39, 98%) although one was poorly lit. Eighty nine percent of the simulations were done where there was adequate space, while 13% were done in crowded areas. Simulation supplies were also usually placed in convenient locations (*n* = 36, 92%). In all cases, the debriefs were conducted in the same location as the simulations.

### Simulation facilitation skills

All the simulations selected for analysis contained the expected postpartum hemorrhage due to atony scenario. In all but 2 of the scenarios, AMANAT-Jyoti NM were the educators of the simulation. Contrary to instructions AMANAT-Jyoti NM were the participants in the remaining 2, NMS were lead educators. Table 3 shows the proportion of videos with observed positive indicators such as use of hand signals and whiteboard to communicate vital signs and negative indicators such as laughing during the simulation scenario. Eventhough Of note, in 73% of the simulated scenarios, simulated blood was present and visible. All simulated patients were to have been coached to express concern for the wellbeing of the baby, however, in only 13% of the scenarios this was shown.

**Table 3:** Video analysis of simulation facilitation skills of AMANAT-Jyoti Nurse Mentors of a postpartum hemorrhage due to uterine atony scenario in Bihar, India from May – August, 2018 (*n* = 39)

Simulation Educator Skills		
Indicator	*N*	% of Videos
Educator role		
Simulation started at an appropriate time	39	100
Used hand signals to communicate with patient actress at least once	2	6
Adjusted blood flow during simulation to enhance realism	0	0
Communicated vital signs via whiteboard with participants	15	38
Did not communicate verbally with participant	39	100
Did not give vitals before provider took them	38	97
Did not communicate verbally with patient	38	97
Physically helped participants during scenario	2	5
Verbally coached participants during scenario	0	0
Participant able to see vital signs on whiteboard	38	95
Simulation Participants		
Asked educator what to do during scenario	1	3
Talked to camera/audience during scenario	3	8
Did not laugh	38	97
Patient Actress/Companion Role		
Wore PartoPants (birth simulator)	39	98
Wore injection pads	16	40
Wore IV arm bands	27	68
Could see the educator	39	98
Did not laugh	39	100
Did not tell participant how to manage the scenario	37	93
PRONTO’s Post-partum hemorrhage due to atony scenario specific		
Patient actress got dizzy	28	70
Baby doll was present	17	43
Patient actress asked for baby	13	33
Blood stains present	29	73
Blood replacement present	2	5
Observers		
Verbally coached simulation participants	3	8
Physically helped simulation participants	0	0

### Debrief facilitation skills

All debrief videos contained Diamond’s three phases debrief: description, analysis and application [12]. The average number of times the educator (i.e. AMANAT-Jyoti Nurse Mentor) spoke was 50 (range: 22 – 100), whereas the participants spoke on average 43 times (range: 17-91) during a debrief. This gave an average 1.2:1 (range of 0.6:1 – 1.7:1) for the ratio of times educators spoke to the times participants spoke. As reference, debriefers are instructed to aim for a 1:1 speaking ratio.

Educators discussed behavioral objectives in 35 videos (90%), ranging from one to three times. They discussed either cognitive or technical objectives in all debriefs, and the number of times ranged from one to five mentions. Table 4 shows the proportion of videos with observed indicators for debrief facilitation (e.g. phases of the debrief, types of learning objectives discussed and formation of the debrief).

**Table 4:** Video analysis of debrief facilitation skills of AMANAT-Jyoti Nurse Mentors of a post-partum hemorrhage due to atony scenario in Bihar, India from May – August, 2018 (*n* = 40)

Debrief Facilitation Skills		
Indicators	*N*	Percentage
1) Educator was sitting	39	100
2) Participants were sitting	39	100
3) Debriefing formation		
Circle	11	28
Semi-circle	21	54
Other	7	18
4) Debrief contributors		
Scenario participants	1	3
Both scenario participants & observers	34	87
Unknown	1	3
5) Simulated patient spoke	24	62
6) Number of participants who spoke individually		
2 participants	11	28
3 participants	13	33
4 participants or more	16	23
		5
		13
7) Number of participants who missed the debrief		
2 participants	2	5
3 participants	2	5
4 participants or more	1	3
8)Educator questions formulation		
Always reading guide	4	10
Mostly reading guide	7	18
Split evenly	20	51
Mostly asking questions	6	15
Always asking questions	0	0

## Discussion

To sustain and continue improving the maternal and newborn health gains in Bihar, India, it is imperative to invest in novel, impactful and long-lasting continuing education methodologies for the local workforce. To our knowledge, this is the first study to evaluate knowledge, attitudes, and simulation facilitation and debriefing skills of a third generation of nurse mentor simulation educators in India, and one of the few studies globally. By analyzing before and after simulation educator training data, we found that the simulation educator training conducted by NMS (second generation educators trained by PRONTO Master Trainers) led to increased simulation facilitation knowledge and attitudes self-assessment among AMANAT-Jyoti NM (third generation educators) in Bihar. Analysis of simulation videos showed that primary health clinics had the appropriate physical infrastructure to conduct simulation training, and that the AMANAT-Jyoti NM had developed basic skills to conduct simulations and debriefs at their assigned clinic. In short, almost 700 nurse mentors were able to perform simulation and debriefing according to simulation training best practice for novice educators [15].

In this assessment, we found that some simulation facilitation skills such as communicating via whiteboard and the use of simulation supplies during simulated scenarios were widely adopted. Similarly, some debriefing practices such as sitting in a semicircle to foster a welcoming environment and delivery of the debrief following the Diamond approach (description, analysis and application) were commonly implemented. However, advanced simulation skills such as rapid adjustment of blood flow and formulation of questions in a constructive manner were observed less frequently. Both findings are consistent with Benner’s novice to expert framework that argues that simulation and debrief facilitation abilities require practice and that competency is achieved after 2 to 3 years of practice and feedback on their facilitation skills from more experienced simulation educators [15]. Notably, despite the common infrastructure challenges in Bihar’s health facilities, all the primary health clinics under study used an appropriate area to conduct simulations. Nurse mentors tended to conduct most of the simulations in classroom settings as opposed to labor rooms, which would be considered the gold standard for in-situ obstetric and newborn emergency simulation training. Most likely, the labour and delivery rooms were occupied with patients when the training took place.

The video analysis showed mixed results in key aspects of the simulation setup and fidelity for the post-partum hemorrhage scenario. For example, 28% of the simulations did not use any fake blood during the scenario as instructed in the simulation guide. The reasons for this could include lack of knowledge on how to make simulated blood, lack of supplies, or lack of understanding about the importance of fake blood for adding to the realism of the scenario. A positive finding was that there were no scenarios in which the patient actress laughed during the scenario. The patient actress role is typically performed by an actual nurse at the facility as opposed to a hired actor, absence of laughter suggests the educators were able to communicate the importance of role play to recreate the emergency and to maintain the seriousness of the scenario throughout.

The close educator to participant speaking ratio was a surprising finding, as novice debriefers tend to exhibit more didactic characteristics and spend more time speaking than participants [16]. This result contrasts with a previous study that argued that in countries such as India with a high-power distance index (PDI) defined as “acceptance of inequality in distribution of power in a certain society” and a measure of hierarchical structures, educators tend to speak for longer periods of time than in countries with a low PDI [17]. This study however did not account for debriefer experience. We hypothesize that novice debriefers in high PDI countries like India are less confident and therefore may tend to speak less than more seasoned debriefers.

We have yet to assess the impact of third generation of training on maternal and neonatal outcomes. However, PRONTO simulation and teamwork trainings in India and elsewhere have been shown to increase the use of evidence-based practices and to increase the identification and management of maternal and newborn complications [18, 5, 19].

## Limitations and strengths

We recognize this study has several limitations including the small number of simulation videos which account for only 11% of the facilities covered under the AMANAT-Jyoti program. We acknowledge this sample may not be representative of all healthcare facilities in Bihar. However, the authors selected diverse facilities to provide a window into the skills of different third generation simulation educators complementing the knowledge and attitudes data. Second, the results in Tables 1 and 2 are not paired due to difficulties in matching individual data. However, given the large sample size (*N* = 701), we think the unpaired results are still illustrative. Lastly, the code window indicators for simulation and debrief were not previously validated and instead developed by a team of experts who have been working in the field of simulation training in low-resource settings.

## Conclusion

Despite limitations, this study is one of the few studies to assess the feasibility of using a third generation of simulation trainers to provide simulation training at scale in a low resource setting such as Eastern India. This study provides evidence that simulation-based training and facilitation concepts are accepted in Bihar by nurse mentors and can be passed on from a first to a second to a third generation of trainers. Additionally, the study showed that the AMANAT-Jyoti program has enabled the public facilities in this resource-poor setting to allocate time and space to conduct in-situ simulations. Lastly, this assessment gives us an understanding of the overall strengths and weaknesses of AMANAT-Jyoti NM’s simulation and debriefing skills and provides important insights and concrete information to provide meaningful feedback to these novice simulation educators. By continuously providing feedback from more experienced mentors, we hope to ensure the sustainability of simulation-based obstetric and newborn care training nurses in Bihar, India. Future work should focus on how to cement simulation facilitation and debriefing skills, maintain educator engagement, and move facilitators towards expert practice in a sustainable manner.

## Data Availability

The datasets generated and/or analysed during the current study are not publicly available due to a memorandum of understanding between our implementing partner and the government of Bihar, India. Data are however available from the authors upon reasonable request and with permission of the government of Bihar.

## Abbreviations

AMANAT: *Aapatkalin Matritva Avam Navjaat Shishu Tatparta* (in Hindi) Emergency Preparedness for Maternal, Newborn and Baby
AMANAT-Jyoti: AMANAT light
BEmONC: Basic Emergency Obstetric and Newborn Care
CEmONC: Comprehensive Emergency Care Obstetric and Newborn
DH: District Hospital
MNMT: Mobile Nurse Mentoring Team
SET: Simulation Educator Training
NMS: Nurse Mentor Supervisor
NM: Nurse Mentor
AMANAT: Aapatkalin Matritva Avam Navjaat Shishu Tatparta.
